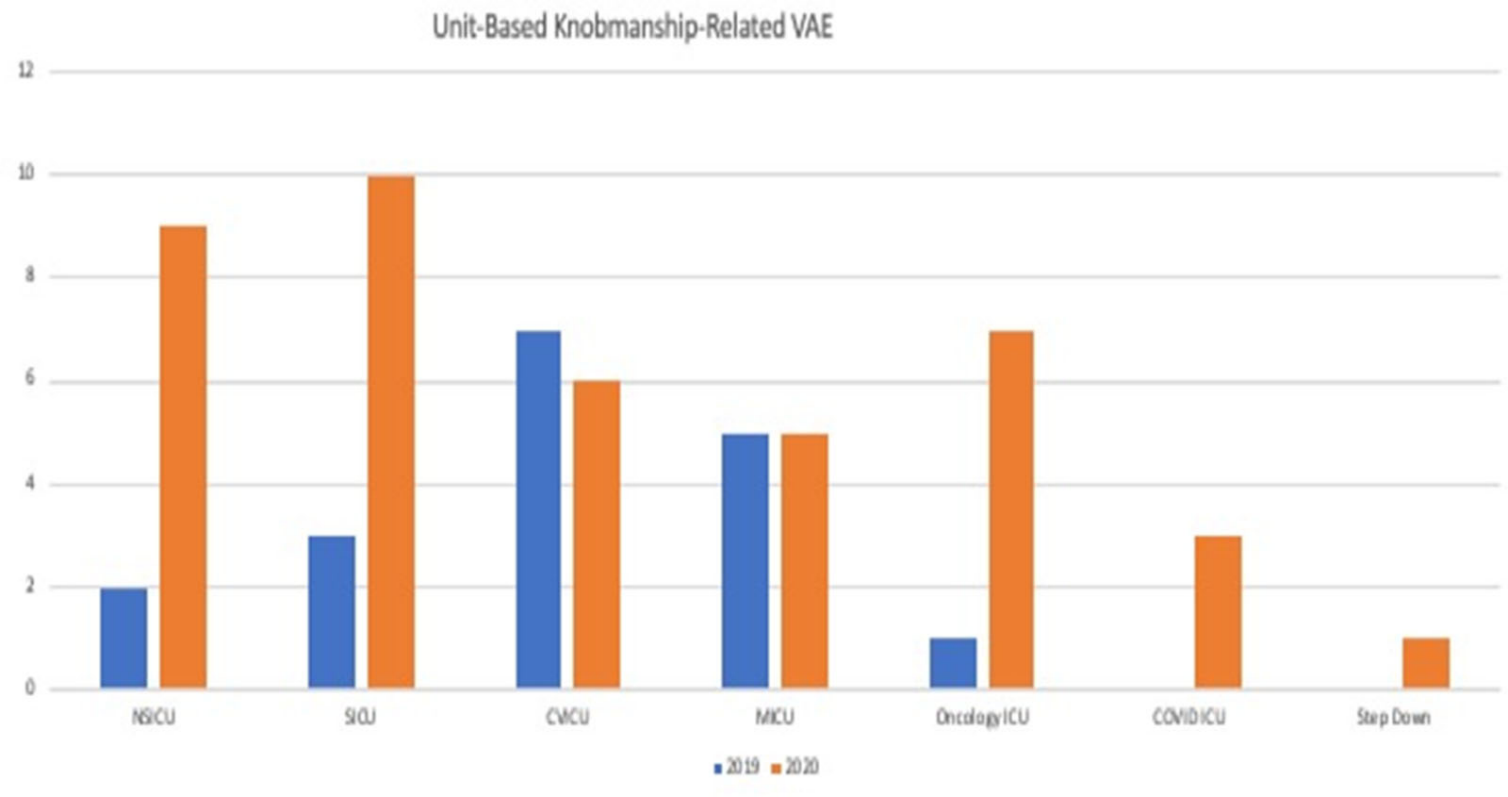# Knobmanship: Dialing Up Understanding of VAE Triggers

**DOI:** 10.1017/ash.2021.158

**Published:** 2021-07-29

**Authors:** Kelly Cawcutt, Mark Rupp, Lauren Musil

## Abstract

**Background:** Mechanical ventilation is a lifesaving therapy for critically ill patients. Hospitals perform surveillance for the NHSN for ventilator-associated events (VAE) by monitoring mechanically ventilated patients for metrics that are generally thought to be objective and preventable and that lead to poor patient outcomes. The VAE definition is met in a stepwise manner; initially, a ventilator-associated condition (VAC) is triggered with an increase in positive end-expiratory pressure (PEEP, >3 cm H_2_O) or fraction of inspired oxygen (FIO_2_, 0.20 or 20 points) after a period of stability or improvement on the ventilator. We believe that many reported VAEs could be avoided by provider and respiratory therapy attention to “knobmanship.” We define knobmanship as knowledge of the VAE definition and trigger points combined with appropriate clinical care for mechanically ventilated patients while avoiding unnecessary triggering of the VAE definition by avoiding small unneeded changes in PEEP or FIO_2_. **Methods:** We performed a chart review of 283 patients who had a reported VAE to the NHSN between January 1, 2019, and December 31, 2020. We collected data including type of VAE, VAE triggering criteria, and clinical course. **Results:** Of the 283 VAEs, 59 were triggered by a PEEP increase from 5 to 8 with stable or decreasing FIO_2_. Of the 59 VAEs, 33 were VACs, 18 were infection-related ventilator- associated complications (IVACs), and 8 were possible ventilator-associated pneumonia (PVAP). Most of these transient changes in PEEP were deemed clinically unnecessary. A 21% reduction of VAEs reported to the NSHN over the 2-year review period could have been avoided by knobmanship. **Conclusions:** The VAE definition may often be triggered by provider bias to the ventilator settings rather than what the patient’s clinical-condition requires. Attention to knobmanship may result in substantial decrease in reported VAE.

**Funding:** No

**Disclosures:** None

**Figure 1.**